# A TabNet-Based Multidimensional Deep Learning Model for Predicting Doxorubicin-Induced Cardiotoxicity in Breast Cancer Patients

**DOI:** 10.3390/cancers18010117

**Published:** 2025-12-30

**Authors:** Juanwen Cao, Xiaojian Hong, Li Dong, Wei Jiang, Wei Yang

**Affiliations:** 1 Department of Cardiology, The Fourth Affiliated Hospital of Harbin Medical University, Harbin 150001, China; 842617@hrbmu.edu.cn (J.C.); 101232@hrbmu.edu.cn (X.H.); 843203@hrbmu.edu.cn (L.D.); 2 Department of Medical Oncology, Cancer Hospital of Dalian University of Technology, Cancer Hospital of China Medical University, Liaoning Cancer Hospital & Institute, No. 44 Xiaoheyan Road, Dadong District, Shenyang 110042, China

**Keywords:** breast cancer, doxorubicin-induced cardiotoxicity, deep learning, TabNet, predictive modeling

## Abstract

**Objective:** To develop and validate an interpretable deep learning model based on the TabNet architecture for predicting doxorubicin-induced cardiotoxicity (DIC) in patients with breast cancer through integration of multidimensional clinical data. **Methods:** This retrospective study included 2034 patients who received doxorubicin-based chemotherapy at The Fourth Affiliated Hospital of Harbin Medical University between January 2021 and December 2023. Clinical, biochemical, electrocardiographic, and echocardiographic parameters were incorporated into six predictive algorithms: logistic regression, decision tree, random forest, gradient boosting machine, XGBoost, and TabNet. Model discrimination, calibration, and clinical utility were assessed using AUC, C-index, calibration plots, and decision curve analysis. Model interpretability was evaluated through attention-based feature importance and SHAP analysis. **Results:** TabNet achieved the best overall predictive performance, with an AUC of 0.86 and a C-index of 0.80 in the validation cohort, demonstrating superior discrimination, calibration, and generalization compared with all baseline models. Decision curve analysis confirmed its higher net clinical benefit across threshold probabilities. The model identified eight dominant predictors—cumulative anthracycline dose, LVEF, QTc interval, lactate dehydrogenase, creatinine, glucose, hypertension, and platelet count—that collectively reflected myocardial contractility, electrophysiological stability, and systemic metabolic stress. Correlation and clustering analyses revealed that high-risk patients exhibited concurrent QTc prolongation, metabolic disturbance, and LVEF decline, defining a distinct cardiometabolic injury phenotype. These findings highlight TabNet’s ability to uncover complex feature interactions while maintaining transparent and clinically interpretable outputs. **Conclusions:** The TabNet-based multidimensional model provides an accurate, stable, and interpretable tool for individualized prediction of doxorubicin-induced cardiotoxicity, supporting early intervention and precision management in breast cancer patients receiving anthracycline therapy.

## Simple Summary

Doxorubicin is a widely used chemotherapy drug for breast cancer, but it can cause heart damage in some patients, which may limit treatment and affect long-term outcomes. Identifying patients at high risk of cardiotoxicity before or during treatment remains challenging. In this study, we developed an interpretable deep learning model based on the TabNet architecture using routinely collected clinical, laboratory, electrocardiographic, and echocardiographic data. The model accurately predicted doxorubicin-induced cardiotoxicity and identified key risk factors related to cardiac function, electrical activity, and metabolic status. This approach may help clinicians recognize high-risk patients earlier and support personalized monitoring and preventive strategies during chemotherapy.

## 1. Introduction

Breast cancer remains the most prevalent malignancy and the leading cause of cancer-related death among women worldwide, accounting for approximately 2.3 million new cases and 670,000 deaths annually, according to GLOBOCAN 2022 estimates [1,2]. Advances in early detection, surgical techniques, and systemic therapies such as chemotherapy, endocrine therapy, and targeted therapy have markedly improved survival outcomes over the past two decades [3,4]. Among these, anthracycline-based regimens, particularly doxorubicin, remain a cornerstone of adjuvant and neoadjuvant chemotherapy for breast cancer, owing to their potent cytotoxic efficacy [5,6,7]. However, the clinical benefit of doxorubicin is counterbalanced by a well-recognized risk of dose-dependent cardiotoxicity, which can lead to irreversible left ventricular dysfunction and heart failure [8,9,10].

Doxorubicin-induced cardiotoxicity (DIC) represents a major dose-limiting complication that significantly impacts long-term prognosis and quality of life in cancer survivors [10]. Despite substantial progress in cardio-oncology, early identification of patients at high risk for DIC remains challenging due to the multifactorial nature of the injury, involving cumulative anthracycline exposure, pre-existing cardiovascular comorbidities, and individual metabolic and genetic susceptibilities [11,12]. Conventional risk stratification tools, which rely on linear regression or single-parameter thresholds such as left ventricular ejection fraction (LVEF) decline, are limited in their ability to capture complex, nonlinear interactions among diverse clinical and biochemical variables [13,14]. Consequently, the development of a more accurate, individualized, and interpretable prediction framework is urgently needed to guide preventive and monitoring strategies for DIC. These limitations highlight the need for data-driven methods capable of capturing the complex, multidimensional relationships underlying DIC.

In recent years, machine learning (ML)-based models have shown substantial promise in cardiovascular risk prediction by leveraging high-dimensional clinical data to uncover nonlinear patterns beyond traditional statistical methods [15]. Algorithms such as random forest, gradient boosting machines (GBMs), and extreme gradient boosting (XGBoost) have achieved notable performance improvements in various oncologic and cardiotoxicity-related applications [16,17,18]. Nevertheless, most conventional ML models remain limited by their “black box” nature and lack of interpretability, hindering clinical trust and real-world adoption [19].

Building on these advances, deep learning enables end-to-end feature learning from complex and high-dimensional data, achieving significant progress in predictive modeling [20]. Traditional fully connected and convolutional architectures often lose interpretability and tend to overfit small or moderate clinical datasets [21]. TabNet, developed in 2020 by Google AI, uses an attention-based neural architecture for tabular data and achieves a strong balance between accuracy, interpretability, and computational efficiency [22]. It applies sequential attention and sparse feature selection to focus on the most informative variables during decision making, providing both high predictive power and a clear explanation of feature contributions at global and individual levels [23]. TabNet also demonstrates a strong capability to integrate data from multiple dimensions, which enhances its predictive accuracy [24]. Therefore, constructing a multidimensional feature model that includes clinical, biochemical, electrocardiographic, and echocardiographic data can improve predictive precision by reflecting the complex pathophysiological mechanisms of cardiotoxicity. Based on this rationale, this study developed and validated a TabNet-based framework that integrates multidimensional patient data for individualized prediction of doxorubicin-induced cardiotoxicity, providing both high accuracy and biological interpretability.

## 2. Materials and Methods

### 2.1. Patients

**Figure 1:**
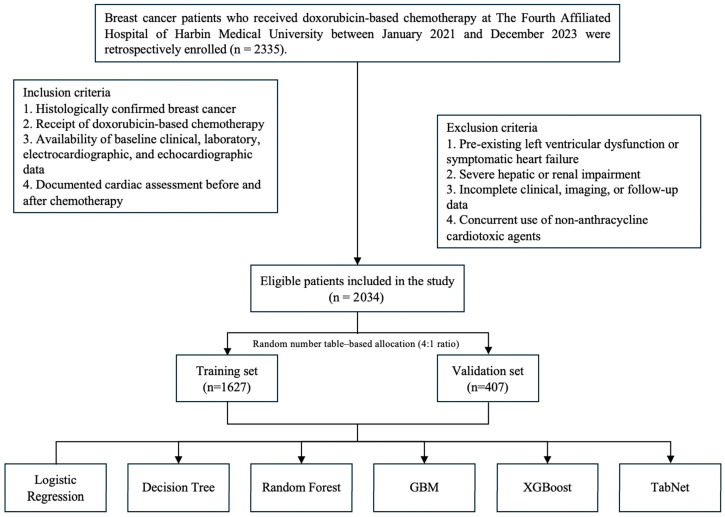
Flow diagram of patient selection and study design.

This retrospective study was conducted at The Fourth Affiliated Hospital of Harbin Medical University and included patients who received doxorubicin-based chemotherapy between January 2021 and December 2023 (Figure 1). A total of 2034 patients with histologically confirmed breast cancer were enrolled after screening, according to predefined inclusion and exclusion criteria. The inclusion criteria were as follows: (1) histologically confirmed diagnosis of breast cancer; (2) completion of at least one full course of doxorubicin-containing chemotherapy; (3) availability of comprehensive clinical, biochemical, electrocardiographic, and echocardiographic data; and (4) documented cardiac follow-up before and after chemotherapy. The exclusion criteria included (1) pre-existing left ventricular dysfunction or symptomatic heart failure prior to chemotherapy; (2) severe hepatic or renal impairment; (3) incomplete or missing clinical, imaging, or follow-up data; and (4) concurrent administration of non-anthracycline cardiotoxic drugs. Based on established diagnostic definitions, all eligible patients were classified into DIC and non-DIC groups for comparative analysis. The study was conducted in accordance with the principles of the Declaration of Helsinki and approved by the Ethics Committee of The Fourth Affiliated Hospital of Harbin Medical University (Approval No. 2024-DWSYLLCZ-15).

### 2.2. Diagnostic Criteria for DIC

To date, there is no universally accepted gold standard for defining DIC. In this study, DIC was identified according to the Chinese Expert Consensus on the Diagnosis and Treatment of Breast Cancer-Related Cardiovascular Diseases (2022 Edition) and the 2022 ESC Cardio-Oncology Guidelines, which provide the most widely recognized framework for clinical and research applications. DIC was defined as new-onset or progressive cardiac dysfunction occurring after doxorubicin exposure that could not be fully explained by other cardiac conditions. Patients were diagnosed with DIC if any of the criteria listed in Table 1 were met.

### 2.3. Data Collection and Preprocessing

Comprehensive multidimensional data were collected from the institutional electronic medical record system of The Fourth Affiliated Hospital of Harbin Medical University. The dataset encompassed four major categories of variables: (1) clinical characteristics, including demographic information, comorbidities, and treatment-related factors; (2) biochemical indicators reflecting hepatic, renal, and metabolic function; (3) electrocardiographic variables describing cardiac electrical activity; and (4) echocardiographic measurements assessing cardiac structure and systolic function. Only patients with complete clinical, laboratory, electrocardiographic, and echocardiographic data were included in the final analysis, according to the predefined inclusion criteria. Therefore, no variable exclusion based on missingness thresholds and no imputation using mean, median, or mode values were performed during the modeling process. Standard preprocessing procedures required for model implementation were applied uniformly across the dataset. Continuous variables were normalized using z-score transformation, and categorical variables were converted into dummy indicators through one-hot encoding.

After preprocessing, the final dataset was randomly divided into a training cohort (*n* = 1627) and an independent validation cohort (*n* = 407) in a 4:1 ratio using stratified random sampling to maintain the proportional distribution of DIC and non-DIC cases across cohorts.

### 2.4. TabNet Architecture

TabNet is a deep learning framework specifically developed for tabular data that integrates sequential attention and sparse feature selection to learn informative feature representations while preserving interpretability. Unlike conventional neural networks that process all input features simultaneously, TabNet employs a feature selection mask at each decision step *i*, defined as

M_i=Sparsemax(P_i W_i)

where P_i represents the prior scale parameter derived from previous attention steps and W_i denotes the learnable weight matrix. The Sparsemax activation constrains the mask values between 0 and 1 and enforces sparsity, allowing the model to focus selectively on the most informative variables at each step. Each decision step transforms the selected features into a latent representation through a nonlinear decision block

d_i=f_i(M_i ⊙ x)

where x is the input feature vector and ⊙ denotes element-wise multiplication. The final prediction output is obtained by aggregating the representations across all decision steps

y=∑_{i=1}^{N} DecisionStep(d_i)

This sequential attention mechanism enables TabNet to dynamically allocate feature importance for each individual sample, achieving interpretable and patient-specific decision pathways. The incorporation of sparse feature selection further minimizes redundancy and mitigates overfitting, thereby enhancing generalization on moderate-sized clinical datasets. Through this design, TabNet not only achieves end-to-end feature selection and interpretability, but also aligns well with the heterogeneity and nonlinear interactions inherent in clinical multidimensional data.

### 2.5. Model Development

To construct a comprehensive predictive framework for DIC, six supervised learning models were developed and systematically compared: logistic regression (LR), decision tree (DT), random forest (RF), GBM, XGBoost, and TabNet. These models were chosen to represent a spectrum of machine learning paradigms, ranging from conventional linear classifiers to ensemble-based and deep learning approaches, thereby enabling a robust comparative evaluation of predictive performance and interpretability. All models were trained using the same dataset, split into a training cohort (80%) and an independent validation cohort (20%) to ensure methodological consistency. Prior to model fitting, all continuous variables were standardized to zero mean and unit variance, and categorical variables were one-hot-encoded. Missing values were imputed using the k-nearest neighbors (KNN) approach. To address potential class imbalance between DIC and non-DIC groups, the synthetic minority oversampling technique (SMOTE) was applied within the training set.

For baseline models (LR, DT, RF, GBM, and XGBoost), hyperparameter optimization was performed using a five-fold cross-validation grid search minimizing the mean squared error for regression-based performance and maximizing the area under the receiver operating characteristic curve (AUC) for classification accuracy. For the TabNet model, optimization was performed through Bayesian hyperparameter tuning, focusing on the learning rate, number of decision steps, relaxation factor (γ), and sparsity regularization coefficient (λ_sparsity). Model training employed an Adam optimizer with an initial learning rate of 0.02, batch size of 512, and early stopping after 30 epochs without improvement in validation loss to prevent overfitting. Model outputs are expressed as predicted probabilities of DIC occurrence. Performance metrics including mean absolute error (MAE), root mean square error (RMSE), coefficient of determination (R^2^), AUC, concordance index (C-index), and stability index (σ) were calculated to assess the predictive accuracy, discrimination, calibration, and robustness of the models. This multi-dimensional evaluation ensured that both global performance and generalization ability were comprehensively characterized.

### 2.6. Statistical Analysis

Statistical analyses were performed using the R software (version 4.3.2) and Python (version 3.10). The statistical analysis framework was designed to describe baseline characteristics, compare group differences, develop and evaluate predictive models, and assess model performance, stability, interpretability, and generalizability. Continuous variables were assessed for normality using the Shapiro–Wilk test and are presented as mean ± standard deviation or median (interquartile range), as appropriate, while categorical variables are expressed as frequencies and percentages. Differences between groups were analyzed using the independent Student’s *t*-test or Mann–Whitney U test for continuous variables and the chi-square or Fisher’s exact test for categorical variables. Multiple predictive models were subsequently developed, and their performance was assessed using complementary metrics reflecting different aspects of predictive performance. Hyperparameter optimization for all models was performed within the training cohort to avoid information leakage. Model discrimination was evaluated using receiver operating characteristic curves, the area under the curve, and the concordance index, while regression performance was quantified using the mean absolute error, root mean square error, and coefficient of determination. Model calibration and clinical utility were assessed using calibration plots, the Hosmer–Lemeshow goodness-of-fit test, and decision curve analysis. Precision–recall curves were obtained to evaluate model robustness under class imbalance. To assess model robustness and reduce the risk of overfitting, model stability and generalizability were systematically examined throughout the modeling process using five-fold cross-validation and 1000 bootstrap resampling. Comparative performance between models was assessed using DeLong’s test for paired AUC comparisons. In addition, subgroup and stratified analyses were conducted according to age, cumulative anthracycline dose, radiotherapy status, and sex to evaluate the consistency of model performance across clinically relevant patient subgroups. All statistical tests were two-sided, and a *p* value < 0.05 was considered statistically significant.

## 3. Results

### 3.1. Patient Characteristics

A total of 2034 patients receiving doxorubicin-based chemotherapy were included in the present study, 305 of which (15.0%) experienced DIC. The mean age of the entire cohort was 54.8 ± 10.5 years, and the mean BMI was 24.9 ± 3.4 kg/m^2^. Compared with patients without DIC, those who developed DIC were significantly older (59.1 ± 9.7 vs. 53.9 ± 10.4 years, *p* < 0.001) and had a higher prevalence of hypertension (59.0% vs. 38.3%, *p* < 0.001), diabetes (30.8% vs. 18.7%, *p* < 0.001), and coronary artery disease (14.8% vs. 8.6%, *p* = 0.004). The distribution of TNM stages differed significantly between the two groups (*p* = 0.030), with stage III and IV disease more common among patients with DIC. Patients who developed DIC also received higher cumulative doses of doxorubicin (311 ± 49 mg/m^2^ vs. 272 ± 53 mg/m^2^, *p* < 0.001) and were more likely to undergo chest radiotherapy (29.6% vs. 21.4%, *p* = 0.007) or HER2-targeted therapy (18.0% vs. 11.4%, *p* = 0.009). No significant difference was found in BMI between the two groups (*p* = 0.068). Collectively, patients with DIC were characterized by older age, more cardiovascular comorbidities, and higher cumulative anthracycline exposure compared with those without DIC (Table 2).

### 3.2. Model Parameters

A total of 2034 patients were randomly divided into a training cohort (n = 1627) and a validation cohort (n = 407). Baseline demographic, clinical, echocardiographic, and laboratory parameters were comparable between the two cohorts, and no significant differences were observed in any variable (all *p* > 0.05). The mean age was 55.0 ± 10.3 years in the training set and 54.6 ± 10.8 years in the validation set, while the mean BMIs were 24.9 ± 3.4 and 25.0 ± 3.5 kg/m^2^, respectively. The prevalence of hypertension, diabetes, and coronary artery disease was similar between cohorts. The mean cumulative dose of doxorubicin was 279 ± 54 mg/m^2^ in the training set and 277 ± 56 mg/m^2^ in the validation set. Cardiac functional indices, including left ventricular ejection fraction, ventricular dimensions, and electrocardiographic parameters, as well as routine biochemical and hematological indicators, showed no significant intergroup differences (all *p* > 0.05). These findings indicate that the training and validation sets were well balanced, providing a consistent foundation for subsequent model construction and validation (Table 3).

### 3.3. Model Development and Performance Evaluation

**Figure 2:**
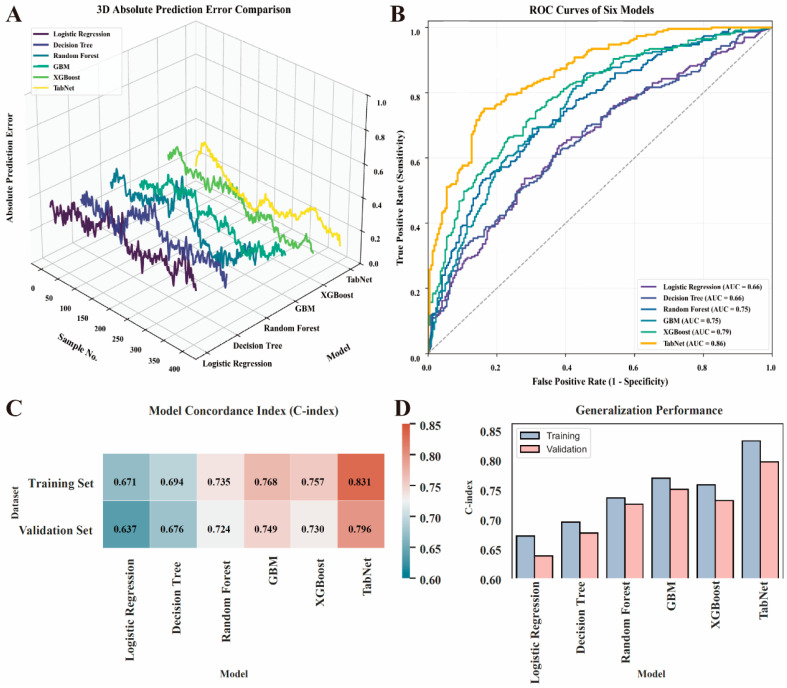
Comparative performance of six predictive models. (**A**) 3D absolute prediction error comparison. (**B**) ROC curves. (**C**) Model concordance indices (C-index heatmap). (**D**) Generalization performance in training and validation cohorts.

Six machine learning models—logistic regression, decision tree, random forest, GBM, XGBoost, and TabNet—were developed to predict doxorubicin-induced cardiotoxicity. Their comparative performance is illustrated in Figure 2A–D. In the 3D absolute prediction error analysis (Figure 2A), TabNet exhibited the lowest overall prediction deviation, indicating better model calibration than other algorithms.

The regression performance metrics of all models are summarized in Table 4. TabNet achieved the lowest mean absolute error (MAE = 0.175) and root mean square error (RMSE = 0.231), together with the highest coefficient of determination (R^2^ = 0.83) and stability index (σ = 0.047). These results demonstrate that TabNet provided superior fitting accuracy and robustness compared with traditional ensemble and linear models.

Receiver operating characteristic (ROC) curve analysis (Figure 2B) further confirmed that TabNet had the strongest classification performance, yielding the largest area under the curve (AUC = 0.86, 95% CI 0.82–0.90), followed by XGBoost (AUC = 0.79) and GBM (AUC = 0.75). The corresponding sensitivity, specificity, and Youden index values for each model are listed in Table 5. TabNet also achieved the best balance between true-positive and true-negative predictions (sensitivity 0.85, specificity 0.78, Youden index 0.63).

The concordance index (C-index) results presented in Figure 2C,D showed consistent trends. TabNet achieved the highest discrimination in both the training cohort (C-index = 0.831) and the validation cohort (C-index = 0.796), reflecting strong generalization performance without overfitting. Taken together, TabNet consistently outperformed all other algorithms across regression, discrimination, and calibration metrics, demonstrating optimal accuracy, stability, and clinical applicability for cardiotoxicity prediction.

To provide an integrated evaluation of predictive performance, a radar chart (Supplementary Figure S1) was constructed to visualize the five key performance dimensions of each model, including AUC, C-index for training and validation cohorts, stability, and generalization ability. As illustrated, TabNet consistently occupied the outermost area across all dimensions, indicating its superior and well-balanced performance profile. Specifically, TabNet demonstrated the highest discrimination ability in both the training and validation cohorts, along with the greatest model stability and generalization capacity. In contrast, traditional models such as logistic regression and decision tree exhibited smaller enclosed areas, reflecting relatively limited discrimination and robustness. These results further confirm that the TabNet framework achieved the optimal trade-off between accuracy, stability, and generalization among all evaluated algorithms.

### 3.4. Model Interpretation

**Figure 3:**
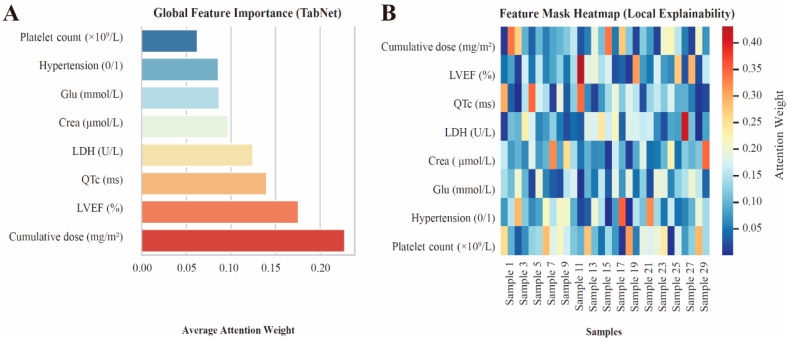
Global and local feature importance of the TabNet model. (**A**) Global attention-based feature importance. (**B**) Feature mask heatmap showing local explainability across samples.

To enhance the interpretability of the TabNet-based prediction model, both global and local feature attribution analyses were conducted. Global feature importance analysis (Figure 3A) revealed that cumulative anthracycline dose, LVEF, and QTc interval were the top three contributors to model output, followed by LDH, creatinine, glucose, hypertension, and platelet count. These features together represent a comprehensive profile encompassing cardiac function, hemodynamic status, and systemic metabolism. The feature mask heatmap (Figure 3B) demonstrated heterogeneous feature weights across individual samples, suggesting that TabNet dynamically adjusted feature attention to achieve personalized risk prediction.

**Figure 4:**
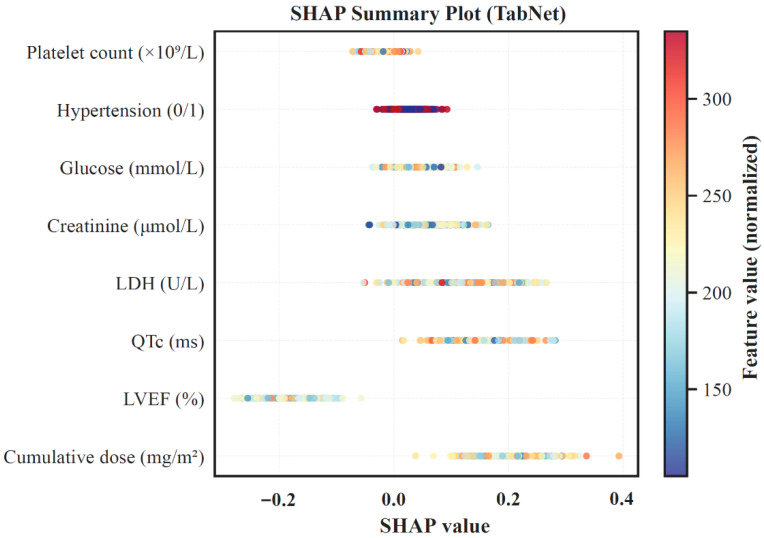
SHAP summary plot of feature contributions in the TabNet model.

To further validate model interpretability, SHAP analysis was performed to quantify the contribution and directionality of each variable (Figure 4). Consistent with the attention-based feature ranking, higher cumulative dose, prolonged QTc, and elevated LDH were positively associated with increased cardiotoxicity risk, whereas higher LVEF exerted a strong protective effect. These findings confirmed the biological plausibility of the TabNet-derived decision process.

**Figure 5:**
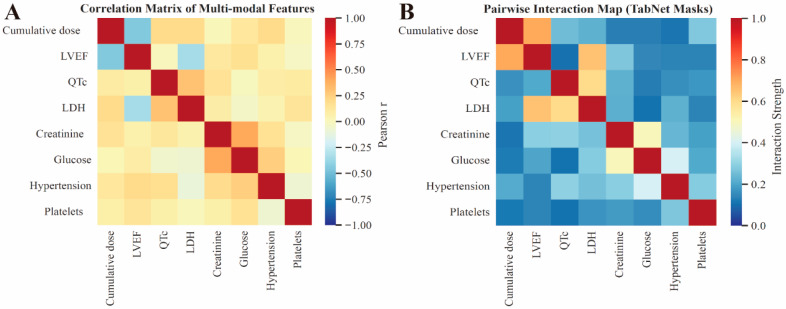
Correlation and interaction analysis of multi-modal features. (**A**) Pearson correlation matrix. (**B**) Pairwise interaction map.

Feature correlation and interaction analyses (Figure 5A,B) provided additional insight into inter-variable relationships. Strong positive correlations were observed between cumulative dose, LDH, and LVEF reduction, whereas QTc prolongation appeared to be moderately linked to metabolic indicators (glucose and creatinine). Pairwise interaction maps derived from TabNet masks revealed that cumulative dose and LVEF formed the most synergistic pair influencing risk prediction, highlighting the central role of anthracycline exposure and cardiac reserve in doxorubicin-induced injury.

**Figure 6:**
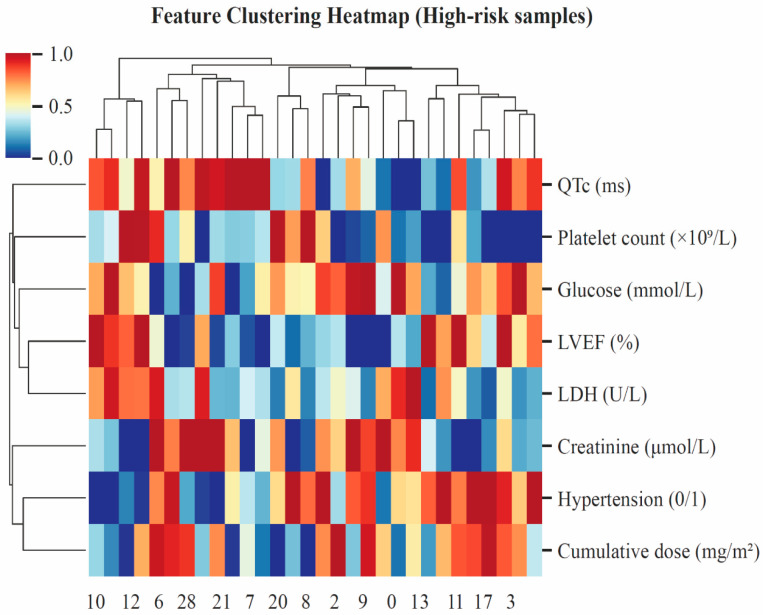
Feature clustering heatmap for high-risk samples.

Finally, hierarchical clustering of high-risk patients (Figure 6) revealed distinct co-activation patterns among cardiometabolic variables. High-risk individuals exhibited a characteristic cluster defined by prolonged QTc, elevated LDH and creatinine levels, and reduced LVEF, indicating concurrent myocardial stress, metabolic disturbance, and contractile dysfunction. Cumulative anthracycline dose and hypertension were positioned within the same cluster, reinforcing their additive contribution to cardiac vulnerability. Collectively, these clustered patterns delineate a cardiotoxic phenotype characterized by metabolic stress, electrophysiological instability, and impaired cardiac function, aligning well with known mechanisms of anthracycline-induced cardiotoxicity.

### 3.5. Model Performance and Clinical Utility Evaluation

To comprehensively evaluate the predictive reliability and clinical applicability of the TabNet model, post hoc performance analyses were conducted using the independent validation cohort. Decision curve analysis (Supplementary Figure S2A) demonstrated that the TabNet model yielded a consistently higher net clinical benefit across a wide range of threshold probabilities compared with the “treat-all” and “treat-none” strategies, indicating favorable clinical usefulness in risk-based decision making. Calibration analysis (Supplementary Figure S2B) revealed close agreement between predicted and observed probabilities, with the calibration curve aligning well along the ideal 45-degree line, suggesting well-calibrated probability estimation without systematic deviation. The precision–recall curve (Supplementary Figure S2C) achieved an AP of 0.54, confirming robust sensitivity and precision despite moderate class imbalance. The predicted probability distribution (Supplementary Figure S2D) further illustrated clear separation between DIC and non-DIC groups, underscoring the model’s strong discriminative capacity. Collectively, these results indicate that the TabNet model maintained stable predictive performance and high clinical interpretability in the independent validation cohort, supporting its potential application for individualized cardiotoxicity risk prediction in anthracycline-treated patients.

### 3.6. Subgroup and Stratified Validation Analysis

To further verify the robustness and clinical generalizability of the TabNet model, stratified and subgroup validation analyses were performed in both the training and validation cohorts. As shown in Supplementary Figure S3A,B, patients were stratified into high-risk and low-risk groups based on the median predicted probability derived from the TabNet model. In both the training and validation cohorts, the high-risk group exhibited a markedly higher cumulative predicted risk of DIC compared with the low-risk group (training cohort *p* < 0.001; validation cohort *p* = 0.022). The clear separation of the two curves highlights the model’s strong discriminative power in distinguishing patients with differing susceptibility to anthracycline-related cardiac injury.

**Figure 7:**
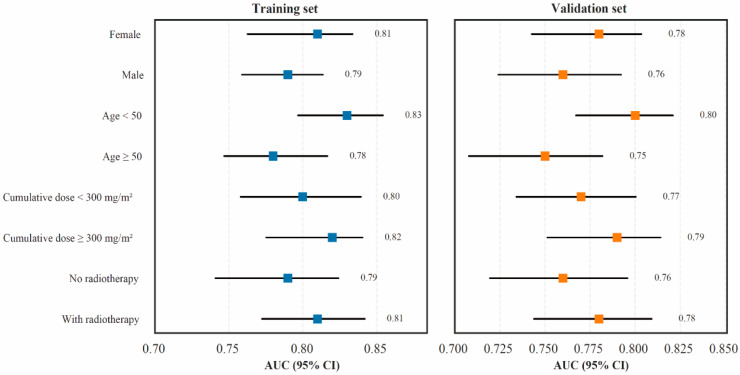
Subgroup analysis of model performance across clinical characteristics.

Subgroup analysis across key clinical characteristics (Figure 7) further confirmed the stability of TabNet’s predictive performance. Consistent AUC values were observed across sex, age, cumulative anthracycline dose, and radiotherapy subgroups, with all AUCs remaining within the range of 0.75–0.83 in both the training and validation sets. Importantly, no single subgroup or individual variable, including age, disproportionately influenced the model’s discriminative performance. These findings indicate that the model maintained reliable discrimination independent of demographic or treatment-related heterogeneity. Collectively, the stratified and subgroup validation results demonstrate that the TabNet model achieved robust and stable predictive capability across different patient subpopulations, reinforcing its clinical applicability for individualized cardiotoxicity risk assessment in real-world settings.

## 4. Discussion

DIC remains one of the most critical barriers to optimizing anthracycline therapy in breast cancer, directly affecting treatment continuity, long-term survival, and quality of life [11]. Despite extensive efforts to identify high-risk patients, early prediction of DIC remains a major unmet need in clinical oncology [25,26]. Conventional risk assessment tools rely primarily on post-treatment monitoring of LVEF or single-parameter thresholds, which fail to detect subclinical injury and underestimate the multifactorial nature of cardiotoxicity [13]. Therefore, developing an accurate and interpretable model capable of integrating heterogeneous patient information is crucial for guiding preventive monitoring and individualized treatment adjustment. In this context, advanced machine learning and deep learning techniques offer new opportunities to improve predictive precision and enable truly personalized risk management.

At present, numerous research groups have focused on elucidating the mechanisms underlying doxorubicin-induced cardiotoxicity, with studies spanning mitochondrial dysfunction, ferroptosis, gut microbiota regulation, metabolic reprogramming, and traditional medicine-based interventions, aiming to attenuate or reverse myocardial injury from multiple perspectives [27,28,29]. Although these studies have yielded important mechanistic insights and proposed several potential therapeutic targets, most remain at the basic or early translational stage, and their applicability in routine clinical practice remains limited. In 2025, Li and colleagues compared single-dose and cumulative-dose doxorubicin administration strategies in murine models to induce acute and chronic cardiotoxicity and evaluated associated survival outcomes. Their findings demonstrated that cumulative exposure rather than single-dose intensity is the primary determinant of cardiac injury severity, and they proposed more standardized and reproducible dosing recommendations for experimental modeling [30]. Beyond mechanistic investigations, the development of predictive models has emerged as another important research direction. In 2025, Singh et al. conducted a systematic review of metabolomics studies and reported consistent metabolic disturbances associated with doxorubicin-induced cardiotoxicity across in vitro, in vivo, and clinical settings, involving amino acid metabolism, energy metabolism, and purine/pyrimidine pathways. These findings highlight the potential value of metabolomics in identifying early biomarkers and enabling risk prediction for DIC [31]. With advances in computational methodologies, machine learning approaches have increasingly been applied in this field. In 2024, Huang and colleagues integrated bioinformatics analyses, machine learning algorithms, and weighted gene co-expression network analysis to identify and validate key genes and immune cell infiltration patterns associated with DIC and subsequently constructed a model with favorable diagnostic and predictive performance, underscoring the potential contribution of multidimensional molecular features to early DIC identification [32]. Subsequently, in 2025, another study developed a machine learning model based on routinely available clinical and hematological parameters from breast cancer patients, combined with synthetic data augmentation techniques, to achieve potential prediction of doxorubicin-related cardiotoxicity [33]. By contrast, deep learning approaches have thus far predominantly been applied to target discovery and drug screening in DIC research. In 2023, Liu et al. established a deep learning-assisted, high-content phenotypic screening platform using zebrafish cardiac function, successfully identifying candidate compounds with cardioprotective effects and revealing a potential mechanism involving modulation of the Keap1–Nrf2 pathway to mitigate doxorubicin-induced myocardial injury. This work demonstrated the promise of artificial intelligence-based methods in drug discovery and functional evaluation for DIC [34]. In the same year, Chen and colleagues developed a deep learning-based high-content screening system to precisely quantify doxorubicin-induced DNA double-strand breaks and to screen candidate compounds with cardioprotective properties, further supporting the feasibility of artificial intelligence techniques for early identification and intervention in doxorubicin-related cardiotoxicity [35].

In the present study, we developed and validated a multidimensional, interpretable deep learning framework based on the TabNet architecture to predict DIC in patients with breast cancer. By integrating clinical, biochemical, electrocardiographic, and echocardiographic data, the model achieved superior predictive performance compared with five conventional algorithms—logistic regression, decision tree, random forest, GBM, and XGBoost. TabNet demonstrated the best overall performance across discrimination, calibration, generalization, and stability metrics, with an AUC of 0.86 and a C-index of 0.80 in the validation cohort. Its probability estimates were well-calibrated and maintained robust discrimination across subgroups defined by age, cumulative anthracycline dose, and radiotherapy exposure. Moreover, TabNet provided clear interpretability through attention-based feature attribution, allowing for the identification of both globally important predictors and individualized risk contributors. Together, these results indicate that our TabNet-based model provides a reliable and clinically meaningful tool for early risk identification in anthracycline-treated patients.

In this study, eight features emerged as the most influential predictors of DIC within the TabNet model: cumulative anthracycline dose, LVEF, QTc interval, LDH, creatinine, glucose, hypertension, and platelet count. These indicators collectively represent an integrated profile of cardiac structure, function, and systemic metabolic status [36,37,38]. Cumulative anthracycline dose was identified as the strongest predictor, consistent with the well-established dose-dependent mechanism of doxorubicin cardiotoxicity [39]. Anthracyclines generate reactive oxygen species (ROS) through redox cycling and iron-mediated Fenton reactions, leading to mitochondrial damage, lipid peroxidation, and myofibrillar disarray [40]. Higher cumulative exposure exacerbates oxidative stress and disrupts sarcomeric integrity, resulting in a progressive decline in contractile function. LVEF, a measure of global systolic performance, naturally reflects myocardial contractility and reserve capacity [41]. In our model, lower baseline LVEF was strongly associated with higher DIC risk, aligning with prior evidence that patients with reduced myocardial strain or borderline ejection fraction are more vulnerable to anthracycline-induced dysfunction [42]. The QTc interval, another key feature, serves as a surrogate marker of electrical instability and ventricular repolarization heterogeneity [43]. Anthracycline exposure has been shown to prolong QTc by modulating ion channel kinetics and disrupting mitochondrial energy homeostasis in cardiomyocytes. Prolonged QTc not only reflects electrophysiological disturbance, but also predisposes patients to malignant arrhythmias, amplifying the clinical impact of subclinical myocardial injury [44]. Metabolic indicators, particularly LDH, creatinine, and glucose, further enhanced the model’s predictive performance by capturing systemic metabolic stress and tissue injury. Elevated LDH represents increased anaerobic metabolism and cellular turnover, often observed in myocardial ischemia or oxidative stress conditions [45]. Creatinine elevation indicates impaired renal clearance, which has been associated with reduced cardiac output and altered drug pharmacokinetics [46], both of which increase the likelihood of anthracycline accumulation and toxicity. Hyperglycemia and insulin resistance contribute to endothelial dysfunction [47], mitochondrial impairment, and inflammation [48], which synergistically exacerbate anthracycline-induced oxidative damage. These metabolic factors may thus act as amplifiers of cardiac vulnerability.

Hypertension and platelet count, though traditionally regarded as secondary factors, emerged as significant contributors within the TabNet framework. Chronic hypertension accelerates myocardial remodeling and increases left ventricular wall stress, thereby lowering the threshold for anthracycline-induced damage [49]. Platelet count reflects vascular and inflammatory homeostasis, while mild thrombocytosis or activation can indicate subclinical inflammation and endothelial dysfunction [50], both of which potentiate cardiotoxicity via microvascular injury. The observed clustering between cumulative dose, hypertension, and metabolic markers in the feature interaction analysis suggests a tightly interconnected network of hemodynamic, metabolic, and oxidative stress pathways underlying DIC development.

Collectively, these eight predictors delineate a biologically coherent and clinically interpretable model that integrates myocardial, vascular, and metabolic domains. This multidimensional interaction pattern highlights that DIC is not a single-organ phenomenon, but rather the manifestation of systemic cardiometabolic stress, which reinforces the necessity of multi-parameter risk assessment.

From a clinical perspective, the proposed TabNet framework holds considerable potential for enhancing precision cardio-oncology. By identifying patients with high predicted DIC risk, clinicians could implement proactive management strategies, such as limiting cumulative anthracycline exposure, substituting with less cardiotoxic analogs, or initiating cardioprotective therapy (e.g., beta-blockers, ACE inhibitors, or dexrazoxane). The model’s interpretability also facilitates clinical adoption, as it enables visualization of individual-level feature contributions and transparent risk explanation, bridging the gap between complex deep learning algorithms and routine clinical decision making. Moreover, its integration of multimodal data—combining biochemical, electrocardiographic, and echocardiographic variables—aligns with the emerging paradigm of holistic patient profiling, which aims to move beyond single-parameter monitoring toward systems-based risk assessment. The application of such interpretable artificial intelligence in real-world practice could ultimately enable personalized chemotherapy planning and improve long-term cardiovascular outcomes in breast cancer survivors.

Despite these strengths, several limitations should be acknowledged. Although the sample size in this study was relatively large, the analysis was conducted using a single-center retrospective design, which may restrict the generalizability of the findings. Although doxorubicin-induced cardiotoxicity was defined according to established national and ESC guidelines, variability in echocardiographic measurements (such as LVEF and GLS), together with differences in biomarker interpretation, may have introduced potential outcome misclassification. Follow-up information was derived from routine clinical records, and incomplete post-chemotherapy follow-up cannot be fully excluded, which may increase the risk of information bias. External validation in multicenter and prospective cohorts is therefore warranted to further confirm the robustness and clinical applicability of the proposed model. As this study represents an early stage of model development, the proposed framework is not intended for direct bedside application and requires prospective validation and further translational development. At the current stage, the model primarily serves as a foundational tool for risk stratification and hypothesis generation rather than immediate clinical deployment. Beyond these considerations, the model was developed using routinely collected clinical and imaging variables without incorporating molecular or longitudinal data, such as genomic or proteomic profiles, which could further enhance biological interpretability and predictive precision. Although the TabNet model demonstrated superior interpretability compared with conventional neural networks, its decision pathways still require prospective validation and integration into clinical workflows to evaluate its feasibility and safety in real-world practice. Looking ahead, future research should focus on developing dynamic modeling strategies that incorporate temporal variations in biomarkers or imaging indices to better characterize the progressive trajectory of anthracycline-induced cardiac injury.

## 5. Conclusions

This study established an interpretable and multidimensional deep learning model based on the TabNet architecture for individualized prediction of doxorubicin-induced cardiotoxicity in breast cancer. By integrating heterogeneous patient data and leveraging attention-based feature learning, the model achieves robust predictive accuracy and biological transparency. This framework not only demonstrates the feasibility of interpretable deep learning in complex clinical prediction, but also provides a practical foundation for precision risk assessment and proactive cardio protection in oncology practice.

In clinical practice, such a predictive framework may facilitate early risk stratification for anthracycline-induced cardiotoxicity and support individualized surveillance and cardioprotective strategies during cancer treatment. More broadly, these findings highlight the growing importance of early and interpretable risk prediction models in cardio-oncology, reflecting how the integration of multimodal clinical data with emerging deep learning approaches can advance individualized risk stratification. By enabling timely identification of patients at heightened risk of anthracycline-induced cardiotoxicity before the onset of overt cardiac dysfunction, this approach supports earlier risk awareness and more informed clinical decision making.

## Figures and Tables

**Table 1:** Diagnostic criteria for DIC.

Parameter	Diagnostic Criteria
Left ventricular ejection fraction (LVEF)	≥10% absolute reduction from baseline to <53% or ≥5% reduction to <53% with symptoms or signs of heart failure.
Global longitudinal strain (GLS)	>15% relative reduction from baseline, indicating subclinical myocardial injury.
Cardiac biomarkers	Persistent elevation of hs-cTnI/T or NT-proBNP above the upper reference limit, or a 1.5- to 2-fold increase from baseline.
Clinical manifestations	New or worsening symptoms of heart failure, such as dyspnea, fatigue, or peripheral edema.

**Table 2:** Patient characteristics.

	Total	DIC	Non-DIC	
Variable	n = 2034	n = 305	1729	*p*
Age (years)	54.8 ± 10.5	59.1 ± 9.7	53.9 ± 10.4	<0.001
BMI (kg/m^2^)	24.9 ± 3.4	25.4 ± 3.5	24.8 ± 3.4	0.068
Hypertension (%)	41.6	59	38.3	<0.001
Yes	41.60%	59.00%	38.30%	
No	58.40%	41.00%	61.70%	
Diabetes (%)	20.5	30.8	18.7	<0.001
Yes	20.50%	30.80%	18.70%	
No	79.50%	69.20%	81.30%	
Coronary artery disease (%)	9.6	14.8	8.6	0.004
Yes	9.60%	14.80%	8.60%	
No	90.40%	85.20%	91.40%	
TNM stage				0.030
Stage I	18.50%	12.00%	19.70%	
Stage II	37.30%	38.50%	37.00%	
Stage III	31.00%	34.00%	30.40%	
Stage IV	13.20%	15.50%	12.90%	
Cumulative dose (mg/m^2^)	278 ± 55	311 ± 49	272 ± 53	<0.001
Chest radiotherapy				0.007
Yes	22.70%	29.60%	21.40%	
No	77.30%	70.40%	78.60%	
HER2-targeted therapy				0.009
Yes	12.50%	18.00%	11.40%	
No	87.50%	82.00%	88.60%	

**Table 3:** Comparison between the training and validation cohorts.

	Training Set	Validation Set	
Variable	n = 1627	n = 407	*p*
Age (years)	55.0 ± 10.3	54.6 ± 10.8	0.472
BMI (kg/m^2^)	24.9 ± 3.4	25.0 ± 3.5	0.682
Hypertension (%)	41.8	40.7	0.613
Yes	41.80%	40.70%	
No	58.20%	59.30%	
Diabetes (%)	20.3	21	0.745
Yes	20.30%	21.00%	
No	79.70%	79.00%	
Coronary artery disease (%)	9.5	10.1	0.699
Yes	9.50%	10.10%	
No	90.50%	89.90%	
TNM stage			0.511
Stage I	18.30%	19.20%	
Stage II	37.50%	36.80%	
Stage III	31.20%	30.40%	
Stage IV	13.00%	13.60%	
Cumulative dose (mg/m^2^)	279 ± 54	277 ± 56	0.594
Chest radiotherapy (%)	22.5	23.1	0.808
Yes	22.50%	23.10%	
No	77.50%	76.90%	
HER2-targeted therapy (%)	12.6	11.8	0.671
Yes	12.60%	11.80%	
No	87.40%	88.20%	
Systolic BP (mmHg)	126 ± 15	127 ± 14	0.513
Diastolic BP (mmHg)	78 ± 9	77 ± 9	0.42
Heart rate (bpm)	76 ± 11	75 ± 10	0.286
QTc (ms)	423 ± 26	424 ± 25	0.661
QRS duration (ms)	93 ± 12	92 ± 13	0.338
LVEF (%)	62.7 ± 6.6	62.9 ± 6.8	0.764
LVEDD (mm)	48.4 ± 4.8	48.3 ± 4.9	0.837
LA diameter (mm)	36.9 ± 4.2	36.7 ± 4.3	0.541
E/A ratio	1.03 ± 0.28	1.04 ± 0.27	0.678
ALT (U/L)	25 [19–32]	25 [18–31]	0.729
AST (U/L)	24 [19–30]	24 [19–29]	0.842
GGT (U/L)	28 [20–39]	27 [20–38]	0.504
LDH (U/L)	211 ± 61	210 ± 60	0.812
TBIL (μmol/L)	12.0 [9.0–15.0]	12.1 [9.2–15.3]	0.905
DBIL (μmol/L)	3.6 [2.8–4.6]	3.6 [2.9–4.7]	0.772
IDBIL (μmol/L)	8.3 [6.3–10.5]	8.3 [6.4–10.6]	0.931
TP (g/L)	69.8 ± 5.1	69.9 ± 5.2	0.876
ALB (g/L)	41.6 ± 3.8	41.8 ± 3.7	0.521
GLB (g/L)	28.2 ± 3.1	28.3 ± 3.0	0.729
A/G	1.47 ± 0.22	1.48 ± 0.22	0.463
PALB (mg/L)	230 [190–270]	231 [192–272]	0.833
BUN (mmol/L)	5.8 ± 1.6	5.8 ± 1.6	0.952
CREA (μmol/L)	73.6 ± 14.1	73.2 ± 13.9	0.74
UA (μmol/L)	341 ± 89	339 ± 90	0.678
ALP (U/L)	85 ± 28	84 ± 27	0.563
GLU (mmol/L)	5.64 ± 1.09	5.61 ± 1.08	0.744
WBC (×10^9^/L)	6.4 ± 1.9	6.3 ± 1.8	0.498
NEU (%)	61 ± 9	61 ± 9	0.882
LYM (%)	28 ± 8	28 ± 8	0.97
MON (%)	7.1 ± 2.0	7.0 ± 2.0	0.512
EOS (%)	2.2 [1.4–3.1]	2.2 [1.5–3.0]	0.789
BASO (%)	0.5 [0.4–0.6]	0.5 [0.4–0.6]	0.946
Hb (g/L)	129 ± 14	129 ± 13	0.944
RBC (×10^12^/L)	4.32 ± 0.48	4.33 ± 0.47	0.807
Hct (L/L)	0.390 ± 0.040	0.391 ± 0.041	0.835
PLT (×10^9^/L)	238 ± 62	239 ± 63	0.884
PT (s)	12.8 ± 2.2	12.9 ± 2.2	0.714
D-dimer (mg/L FEU)	0.32 [0.21–0.51]	0.32 [0.20–0.50]	0.921

**Table 4:** Regression performance metrics of six machine learning models.

Model	MAE	RMSE	R^2^	Stability (σ)
Logistic Regression	0.386	0.472	0.58	0.091
Decision Tree	0.334	0.419	0.63	0.085
Random Forest	0.326	0.401	0.68	0.076
GBM	0.24	0.315	0.74	0.062
XGBoost	0.246	0.308	0.75	0.059
TabNet	0.175	0.231	0.83	0.047

**Table 5:** Classification performance of six predictive models.

Model	AUC	95% CI	Sensitivity	Specificity	Youden Index
Logistic Regression	0.66	0.62–0.70	0.68	0.64	0.32
Decision Tree	0.66	0.61–0.71	0.67	0.63	0.30
Random Forest	0.75	0.71–0.79	0.78	0.72	0.50
GBM	0.75	0.70–0.79	0.80	0.71	0.51
XGBoost	0.79	0.75–0.83	0.82	0.74	0.56
TabNet	0.86	0.82–0.90	0.85	0.78	0.63

## Data Availability

The datasets generated during and/or analyzed during the current study are available from the corresponding author upon reasonable request.

## Supplementary Materials

The following supporting information can be downloaded at: https://www.mdpi.com/article/10.3390/cancers18010117/s1, Figure S1: Overall Performance Comparison of Predictive Models; Figure S2: Comprehensive performance and clinical utility evaluation of the TabNet model; Figure S3: Cumulative predicted risk curves in training and validation cohorts.

## Abbreviations

The following abbreviations are used in this manuscript:

Abbreviation	Full term
ACE	Angiotensin-converting enzyme
Adam	Adaptive moment estimation
A/G	Albumin-to-globulin ratio
ALB	Albumin
ALP	Alkaline phosphatase
ALT	Alanine aminotransferase
AP	Average precision
AST	Aspartate aminotransferase
AUC	Area under the receiver operating characteristic curve
BASO	Basophils
BMI	Body mass index
BP	Blood pressure
BUN	Blood urea nitrogen
CI	Confidence interval
CREA	Creatinine
C-index	Concordance index
DBIL	Direct bilirubin
DCA	Decision curve analysis
DIC	Doxorubicin-induced cardiotoxicity
DT	Decision tree
EOS	Eosinophils
ESC	European Society of Cardiology
GBM	Gradient boosting machines
GGT	Gamma-glutamyl transferase
GLB	Globulin
GLS	Global longitudinal strain
GLU	Glucose
Hb	Hemoglobin
Hct	Hematocrit
hs-cTnI/T	High-sensitivity cardiac troponin I/T
IDBIL	Indirect bilirubin
IQR	Interquartile range
KNN	k-nearest neighbor
LA	Left atrial
LDH	Lactate dehydrogenase
LR	Logistic regression
LVEDD	Left ventricular end-diastolic diameter
LVEF	Left ventricular ejection fraction
LYM	Lymphocytes
MAE	Mean absolute error
ML	Machine learning
MON	Monocytes
NEU	Neutrophils
NT-proBNP	N-terminal pro–B-type natriuretic peptide
PALB	Prealbumin
PLT	Platelet count
PT	Prothrombin time
QTc	Corrected QT interval
QRS	QRS complex duration
RBC	Red blood cell count
RF	Random forest
RMSE	Root mean square error
ROC	Receiver operating characteristic
ROS	Reactive oxygen species
SHAP	SHapley Additive exPlanations
SMOTE	Synthetic minority oversampling technique
TabNet	Tabular Neural Network
TBIL	Total bilirubin
TNM	Tumor–Node–Metastasis staging system
TP	Total protein
UA	Uric acid
WBC	White blood cell count
XGBoost	Extreme gradient boosting
