# Supplementary material for: A TabNet-Based Multidimensional Deep Learning Model for Predicting Doxorubicin-Induced Cardiotoxicity in Breast Cancer Patients
Source: Cancers (Basel). 2025 Dec 30;18(1):117. doi: 10.3390/cancers18010117 (PMC12784847; doi:10.3390/cancers18010117)
Supplement: Supplementary file 1 [file cancers-18-00117-s001.zip › cancers-3997125-supplementary.pdf]

Supplementary Materials

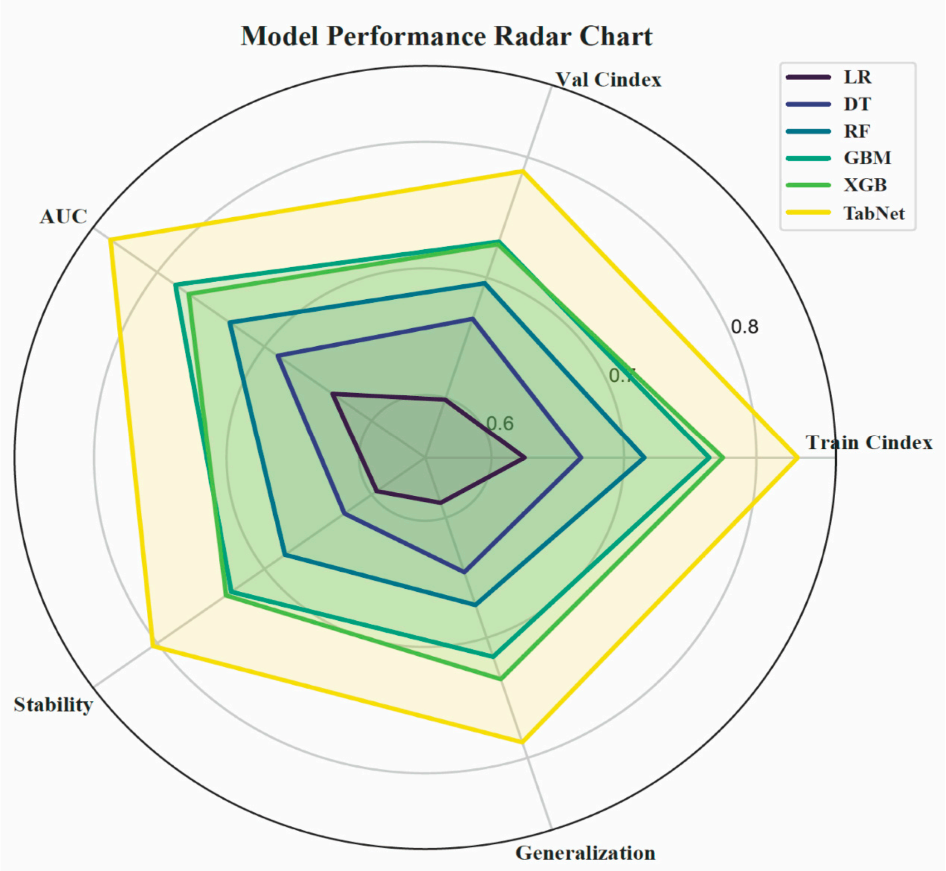

Figure S1. Overall Performance Comparison of Predictive Models.

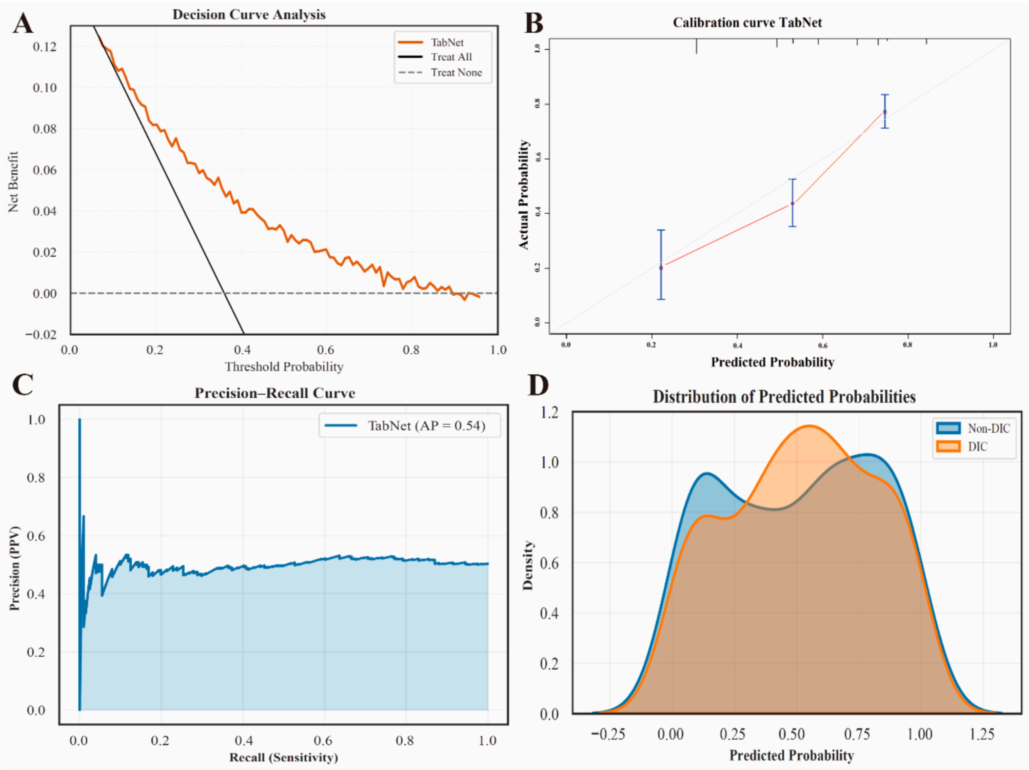

Figure S2. Comprehensive performance and clinical utility evaluation of the TabNet model. (A) Decision curve analysis. (B) Calibration curve. (C) Precision–recall curve. (D) Distribution of predicted probabilities.

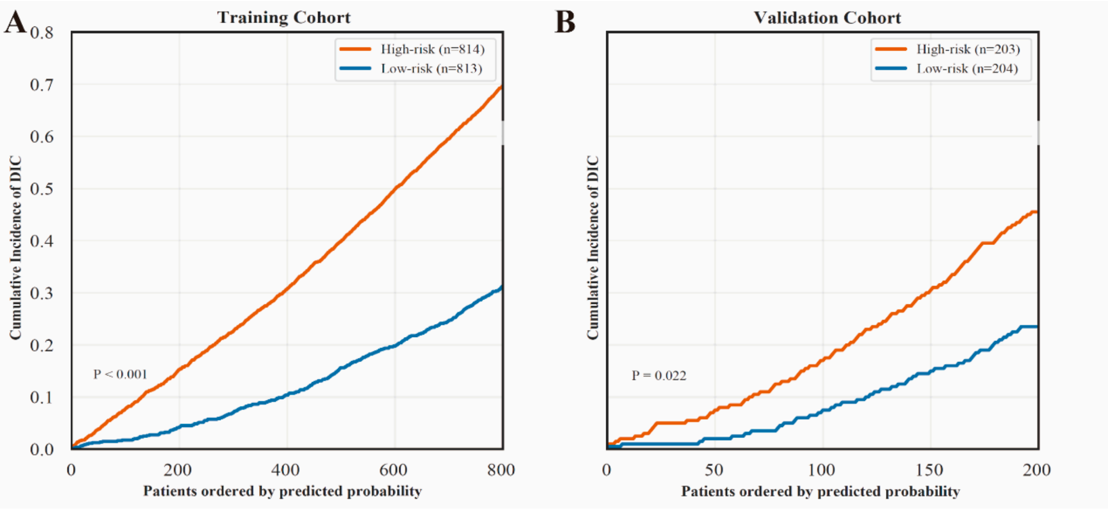

Figure S3. Cumulative predicted risk curves in training and validation cohorts. (A) Training cohort. (B) Validation cohort.
